# Human papillomavirus vaccine initiation and up-to-date vaccine coverage for adolescents after the implementation of school-entry policy in Puerto Rico

**DOI:** 10.1371/journal.pgph.0000782

**Published:** 2022-11-08

**Authors:** Vivian Colón-López, Pamela C. Hull, Olga L. Díaz-Miranda, Mark Machin, Idamaris Vega-Jimenez, Diana T. Medina-Laabes, Roxana Soto-Abreu, María Fernandez, Ana P. Ortiz, Erick L. Suárez-Pérez

**Affiliations:** 1 Department of Cancer Control and Population Sciences, University of Puerto Rico Comprehensive Cancer Center, San Juan, Puerto Rico; 2 Department of Health Services Administration, Graduate School of Public Health, University of Puerto Rico Medical Sciences Campus, San Juan, Puerto Rico; 3 Department of Behavioral Science, College of Medicine, Markey Cancer Center, University of Kentucky, Lexington, Kentucky, United States of America; 4 Department of Health Promotion and Behavioral Sciences, University of Texas Health Science Center, Houston, Texas, United States of America; 5 Department of Epidemiology and Biostatistics, Graduate School of Public Health, University of Puerto Rico, Medical Sciences Campus, San Juan, Puerto Rico; University of the Witwatersrand, SOUTH AFRICA

## Abstract

The human papillomavirus (HPV) vaccine has been proven effective in the prevention of infection with high-risk HPV types, which can lead to the development of six HPV-related cancers. Puerto Rico (PR) adopted a mandatory HPV vaccination school-entry policy that took effect in August 2018. While school-entry requirements are generally accepted as an effective approach for increasing vaccination rates, there are few studies that have documented their impact on improving HPV vaccination rates. The objective of this study was to evaluate the impact of the HPV school-entry policy in PR on HPV vaccine coverage. We used a pre-post natural experiment. The study population included adolescents registered in the PR Immunization Registry during 2008–2019. We calculated HPV vaccine initiation and up-to-date (UTD) vaccine coverage rates. We estimated age-standardized rates (ASR) and standardized rate ratio with 95%CI. Vaccine data corresponding to a total of 495,327 adolescents were included for analysis; 50.9% were male and 49.1% were females. After policy implementation, a marked increase in raw HPV vaccine initiation among 11- to 12-year-old adolescents was observed across years 2017 (a pre-policy year), 2018, and 2019 (58.3%, 76.3%, and 89.8%, respectively). UTD coverage also showed a moderate increase after policy implementation among 11- to 12-year-old adolescents. The gap between sexes in vaccine initiation and UTD coverage narrowed over time; the ASRs in 2019 showed an increase of 19% in initiation and 7% increase in UTD relative to 2017 for males and females combined (both significant at p<0.05). This study demonstrated evidence of improvement in HPV vaccination rates following implementation of the school-entry policy and a narrowed sex gap in vaccine rates over time in PR. Future analyses should assess how the policy continues to affect vaccine coverage in subsequent years and how the COVID-19 pandemic has impacted HPV vaccination uptake.

## Introduction

The human papillomavirus (HPV) vaccine has demonstrated effectiveness in the prevention against infection with high-risk HPV types, which can lead to HPV-related cancers [1–6]. Achieving optimal HPV vaccination uptake is a public health priority [7]. The United States (U.S.) Advisory Committee on Immunization Practice (ACIP) has recommended routine HPV vaccination for 11-to 12-year old females since 2006 and males since 2011 [8]. ACIP recommends a 2-dose schedule for adolescents who initiate HPV vaccine series between 9 through 14 years of age and a 3-dose schedule for adolescents who initiate 15 years or older [8]. Despite strong evidence of its safety and efficacy [9–11], HPV vaccine initiation (having at least 1 dose) coverage is low in the U.S. [12]. In addition, HPV up-to-date (UTD) vaccine coverage rates (all vaccine recommended doses) are lower than Healthy People 2030 goal (80% in females and males) [12, 13].

To address low HPV vaccination coverage rates, five states and territories/jurisdictions in the U.S. have adopted a school-entry requirement for HPV vaccine (Virginia in 2008; the District of Columbia in 2009; Rhode Island in 2015; Puerto Rico (PR) in 2018 and Hawaii in 2020) [14–16]. The efforts made to implement the policy in Puerto Rico and how it was enacted can be found elsewhere [17, 18]. However, policies in these five states/territories are different [19]. The requirements in both Virginia and the District of Columbia have allowed parents to choose to exempt their child from the vaccination requirement for medical, religious, or *philosophical reasons*, which, in effect, made the vaccine optional. In contrasts, Rhode Island, PR, and Hawaii use a more narrow definition for exemptions, allowing parents to exempt their child only for medical or religious reasons [14, 15]. Another essential difference between requirements is the number and timing of doses required to comply with the mandate. Virginia requires two doses of HPV vaccine to students entering seventh grade; the first dose must be administered before the child enters grade [20]. Rhode Island also requires two doses of HPV vaccine for school entry [21]. Adolescents must initiate series before entering seventh grade, and upon entering ninth grade, students must have received the second dose [21]. District of Columbia requires that adolescents entering sixth-to twelve grades with at least 11 years of age must be immunized by the corresponding doses of the HPV vaccine (2 or 3 doses according to the age of first dose receipt) [22]. In Puerto Rico, one dose was required for adolescents 11 to 12 years to comply with the mandate at first implemented [23]. Since the 2021 to 2022 school year, teens from 11 to 16 years must be immunized with at least one dose and are required to complete their series of recommended doses to comply with the mandate [24]. Hawaii requires two doses to adolescents ages 9 through 14 years at first dose; adolescents 15 years or older at first dose are required to complete three doses of HPV vaccine [16]. All new enterers between the seventh and twelve grades must evidence immunizations to corresponding doses according to their age [16].

In general, school-entry-policies have shown to be an effective tool for increasing childhood vaccination rates [14, 25, 26]. However, only a handful of studies have measured their impact on HPV vaccination uptake, with mixed results [14, 27–30]. A study using data from the National Immunization Survey-Teen (NIS-Teen) [27] showed similar trends of HPV vaccination between Virginia and South Carolina in the observed period, and the difference-in-differences in the vaccinated proportions comparisons between the Virginia-South Carolina and the Virginia-Tennessee were not significant. Therefore, there was no evidence of vaccination increase associated with Virginia’s policy [27]. Using NIS-Teen data from 2009–2013, Perkins and colleagues [28] reported female adolescents completion rates increased by nearly 10% in states/jurisdictions with an HPV vaccine school entry mandate (District of Columbia and Virginia), states/jurisdictions with education mandates (Louisiana, Michigan, Colorado, Indiana, Iowa, Illinois, New Jersey, North Carolina, Texas, Washington) and states without mandates during 2009–2013 for all type of states, thus not demonstrating evidence of an increase in the completion rates associated with the implementation of the policy. Another study [14] evaluated NIS-Teen from 2010–2016 and reported a significant (11%) increase in HPV series initiation among boys in Rhode Island one year after enactment of the HPV policy compared with other states with no HPV policy enacted. A significant increase was not observed among girls, although initiation rates among girls were consistently high throughout the observed period [14].

To date, no published studies have analyzed the impact of the HPV vaccine school-entry policies in PR. In addition, the previous studies mentioned that all used NIS-Teen, which is a survey-based data. The majority of U.S. states and territories have an Immunization Information System (IIS), a population-based immunization registry that can be used to generate population-level vaccination coverage rates [31–33]. Further examination of HPV vaccination patterns after implementation of these policies is imperative to inform future efforts to continue improving HPV vaccination through legislation or other strategies. Using the data from the PR Immunization Registry (PRIR), this study aimed to examine the impact of the school-entry requirement in PR that took effect in August 2018 on HPV vaccine initiation and HPV UTD vaccine coverage rates during the first two years of implementation.

## Methods

### The HPV-PIVac Study

This study was part of a larger study entitled *Implementation of School-Entry Policies for Human Papillomavirus Vaccination (HPV-PIVac Study R01CA232743)*. The *HPV PIVac Study* is an ongoing study conducted at the *University of Puerto Rico Comprehensive Cancer Center*. The larger study’s specific aims are: (1) to evaluate barriers and facilitators for the implementation of the HPV vaccine school entry policy in PR, (2) to evaluate the impact on the population’s vaccination coverage, as well as (3) to understand the geographic variation in dissemination, implementation, and results of HPV vaccine school entry policies in USA states and territories. The current analysis reports on findings from aim 2. This study was approved by the *Institutional Review Board* of the *University of Puerto Rico Medical Science Campus* (Protocol number: A8060218).

### Study design

A pre-post natural experiment design was used to assess the impact of school-entry policy on vaccination rates in PR during the first two years of implementation. Since 2018, every August (prior to the start of school), students in PR have been required to show proof that they have received at least one dose of the HPV vaccine if the mandate applies according the adolescents’ age. Students who are not compliant on the first day are given a grace period of 60 days to obtain the vaccine [15]. We assessed the first two years following implementation of the policy.

PRIR IIS was operational from 1994 to 2020 [34] (since the end of 2020, a new registration system was implemented [35]) The primary purpose of an IIS is to record all vaccinations delivered to the population, including dates of vaccination and demographic information. Healthcare providers in vaccinating clinics in PR were ordered by Administrative Order No. 262 of July 18, 2009, to report the administration of vaccines to this registry this order became Law No. 169 on December 12, 2019 [34]. Data from PRIR has strong coverage representing 87% of the Puerto Rican population [36].

### Study population

The study population included adolescents registered in the PRIR during the observation period that were immunized against any or all of the following vaccines: HPV, influenza (Flu), influenza A (H1N1), tetanus, Td (Tetanus, Diphtheria), Tdap, meningococcal conjugate (MenACWY), serogroup B meningococcal (MenB). The inclusion criteria consisted of adolescents in PRIR who were between the ages of 11–17 years as of December 31 of each year from 2008 to 2019. Data from adolescents aged 13 to 17 years was used as a comparison group. It will also allow us to compare with the findings of the NIS-Teen Survey. Exclusion criteria consisted of vaccine doses given being outside the target age range or observed time period (2008–2019) and not being registered or active (individuals not vaccinated at all) in PRIR during this time period.

### Measures

The variables obtained from PRIR included each individual’s unique identifier, date of birth, date of vaccination, HPV vaccine administered, and sex of the adolescent at birth. We used methodology recommended by the American Immunization Registry Association for assessing vaccination coverage using an IIS [31]. We used the ACIP recommended immunization schedule for adolescent vaccines [8] to operationalize two measures of HPV vaccination: HPV vaccine initiation coverage (≥1 dose) and UTD vaccine coverage (receipt of all recommend doses). Recommended HPV vaccine doses was defined as adolescents with three or more doses, and adolescents with two doses when the first HPV vaccine dose was administered before fifteen years old and there was at least five months minus four days between the first and second dose, based on the definition used by the U.S. Centers for Disease Control and Prevention [37]. We used point-in-time assessment method used by the American Immunization Registry Association for age in years as of a specific date each year (i.e., December 31 of each year) for consistency across years. The formulas specifying numerators and denominators are listed below:

$$HPV\;Vaccine\;Initiation\;Coverage=\;\frac{X}{N}*100$$

$$HPV\;UTD\;Vaccine\;Coverage=\;\frac{Y}{N}*100$$

*Where: X* = *Persons in certain age range with at least 1 dose as of Dec 31 per year*

*Y* = *Persons in certain age with recommended doses as of Dec 31 per year*

*N* = *Population in certain age range as of Dec 31 per year*

We calculated these measures for all adolescents aged 11–17 years old, and, separately for younger (11–12 years) and older (13–17 years) adolescents. We also calculated rates for males and females separately and combined.

### Statistical analysis

Descriptive statistics, including patient frequency and percentage, were performed to describe trends in the unadjusted coverage percentages. Understanding that any population change could affect interpretations in coverage analysis [31], we calculated age-standardized rates (ASR) for HPV vaccine (per 100 individuals) using the direct-standardization method with the US 2000 census data distribution [38] as the reference population to control for the effect of the migration in PR in the last years [39]. In the middle of the transition for the implementation of this mandate in Puerto Rico, two atmospheric events, Hurricane Maria and Irma, caused the two great net migration waves in 2017 (n = 77,000, 2.5% of the total population of Puerto Rico) and 2018 (n = 113,000, 3.5% of the total population of Puerto Rico) [40, 41]. For this analysis, the reference year used was 2017, a year prior to the establishment of the policy. The age-standardized rate ratio (SRR) was estimated with 95%CI in order to compare the age-standardized vaccine initiation rate before and after the implemented policy as follows [42]:

$$SRR_{k\;vs.\;\;2017}=\frac{ASR_{k}}{ASR_{2017}}$$

ASR_k_ indicates the age-standardized rate in any other year, different than 2017.

If SRR >1, this would indicate that the ASR_k_ was higher than the ASR_2017_; so, in this condition we would refer to it as an increase in the vaccination rate after the policy took effect. If SRR< 1, this would indicate that the ASR_k_ was lower than the ASR_2017_; so, in this condition, we would refer to it as a reduction in the vaccination rate after the policy took effect. All rates were calculated overall and stratified by age group (11–12 years and 13–17 years) and biological sex as potential predictor variables. Statistical analyses were conducted using Stata version 13 (USA: StataCorp LLC).

## Results

The datafile provided by PRIR contained a total of 3,066,098 records for vaccine doses administered to adolescents between January 1, 2008, and December 31, 2019. A total of 924,596 records for vaccine doses in the database were dropped from this analysis due to being duplicate doses, doses administered prior to the observed period, or additional doses beyond the recommended number of doses per vaccine. As a result, our final database consisted of 2,141,502 records of vaccine doses administered, which corresponded to 495,327 adolescents included for this analysis. Of the total number of adolescents, 252,343 (50.9%) were male and 242,984 (49.1%) were females.

**Fig 1:**
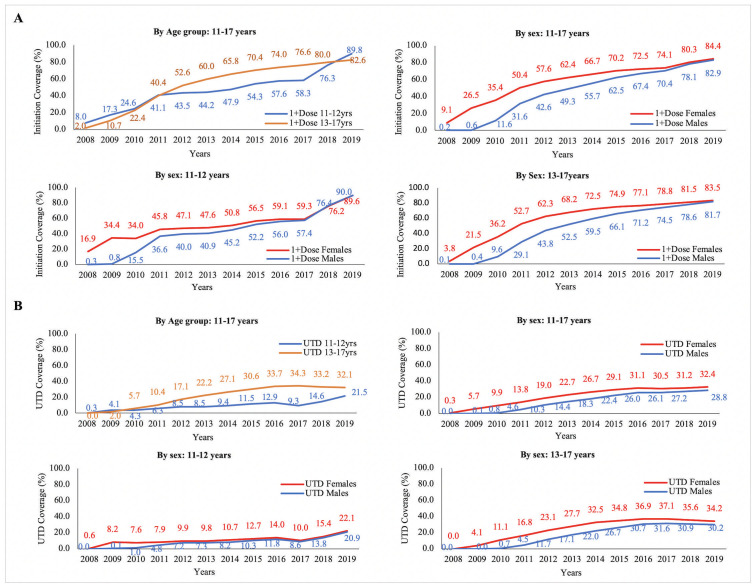
HPV vaccine coverage rates stratified by age group and sex from 2008 to 2019. (A) HPV Vaccine Initiation Coverage Rates, (B) HPV Vaccine UTD Coverage Rates.

Fig 1A shows HPV vaccine initiation coverage (1+Dose) stratified by age group and sex from 2008 to 2019. After policy implementation, a marked increase in HPV vaccine initiation coverage rates among adolescents aged 11 to 12 years was observed; 58.3%, 76.3%, and 89.8% in years 2017, 2018, and 2019, respectively. HPV vaccine initiation by sex shows how the gap between the sexes closed over time. At the end of the period, the difference in initiation rates between the males and females is minimal. This trend is also observed in initiation rates by sex in each age category.

Fig 1B shows HPV vaccine UTD coverage (all recommended doses) stratified by age group and sex from 2008 to 2019. UTD vaccine coverage rates in panel B (see Fig 1) also showed an increase after policy implementation among adolescents 11 to 12 years old, but the increase was not as steep as for initiation coverage rates (9.3%, 14.6% and 21.5% in 2017, 2018 and 2019). For comparison, from 2008 to 2016, rates fluctuated between 0.3% to 12.9%. UTD vaccine coverage rates by sex also showed an elimination of the sex gap for UTD vaccine coverage for 11-and 12-year-old. For 13 to 17-year-old the gap was reduced but not eliminated.

Table 1 reports the ASRs and SRRs (relative to pre-implementation reference year of 2017) for HPV vaccine initiation rates. The ASRs show an increase in HPV vaccine initiation rates of 11% for 2018 and an increase of 19% in 2019, compared to the reference year of 2017 (2018 SRR: 1.11, 95% CI: 1.09, 1.11; 2019 SRR: 1.19, 95% CI: 1.18, 1.20). The same pattern was observed in the analysis stratified by sex, but males started lower and had greater increases than females. The ASRs and SRRs also indicate the gradual historical increasing trend in HPV vaccine initiation for all years from 2008 to 2017 before the policy was implemented. We included S1 File with the distribution of the population stratified by age and sex throughout the study period.

**Table 1:** Age-standardized rates (ASR) (per 100) and standardized rate ratio (SRR) for HPV vaccine initiation among adolescents 11–17 years old in Puerto Rico (2008–2019).

Years	Overall		Females		Males	
	ASR	SRR^a^	ASR	SRR^a^	ASR	SRR^a^
2008	3.70	0.05 (0.05–0.05) ^b^	7.56	0.10 (0.10–0.11) ^b^	0.13	0.00 (0.00–0.00)
2009	12.60	0.18 (0.17–0.18) ^b^	25.24	0.34 (0.34–0.35) ^b^	0.52	0.01 (0.01–0.01) ^b^
2010	23.05	0.32 (0.32–0.33) ^b^	35.55	0.49 (0.48–0.49) ^b^	11.29	0.16 (0.16–0.16) ^b^
2011	40.63	0.57 (0.56–0.57) ^b^	50.69	0.69 (0.68–0.70) ^b^	31.29	0.45 (0.44–0.45) ^b^
2012	49.98	0.70 (0.70–0.71) ^b^	57.55	0.79 (0.78–0.80) ^b^	42.68	0.61 (0.61–0.62) ^b^
2013	55.46	0.78 (0.77–0.78) ^b^	62.27	0.85 (0.84–0.86) ^b^	49.14	0.71 (0.70–0.71) ^b^
2014	60.66	0.85 (0.84–0.86) ^b^	66.22	0.90 (0.90–0.91) ^b^	55.37	0.80 (0.79–0.80) ^b^
2015	65.76	0.92 (0.92–0.93) ^b^	69.61	0.95 (0.94–0.96) ^b^	62.08	0.89 (0.88–0.90) ^b^
2016	69.28	0.97 (0.96–0.98) ^b^	71.88	0.98 (0.97–0.99) ^b^	66.80	0.96 (0.95–0.97) ^b^
2017	71.33	1.00 (Reference)	73.19	1.00 (Reference)	69.54	1.00 (Reference)
2018	78.93	1.11 (1.09–1.11) ^b^	79.95	1.09 (1.08–1.10) ^b^	77.95	1.12 (1.11–1.13) ^b^
2019	84.66	1.19 (1.18–1.20) ^b^	85.24	1.16 (1.15–1.18) ^b^	84.11	1.21 (1.20–1.22) ^b^

^a^ The ratio of each year ASR to 2017 ASR as reference, with 95% confidence interval in parentheses.

^b^ P-value < 0.05

Table 2 reports the ASRs and SRRs (relative to pre-implementation reference year of 2017) for HPV UTD vaccine rates. The ASRs indicated a significant 3% increase in UTD vaccine rates in 2018 (SRR: 1.03, 95% CI: 1.02, 1.04), and 7% increase in HPV UTD vaccine rates for 2019 (SRR: 1.07, 95% CI: 1.06, 1.09) compared to the pre-implementation year 2017. Again, males started lower and displayed a greater magnitude of increase than females. Similar to vaccine initiation, for almost all years before policy implementation, UTD ASRs gradually increased, although more slowly than the vaccine initiation ASRs, then they are plateauing in 2016 and 2017 before increasing again in 2018.

**Table 2:** Age-standardized rates (ASR) (per 100) and standardized rate ratio (SRR) for HPV vaccine UTD among adolescents 11–17 years old in Puerto Rico (2008–2019).

Years	Overall		Females		Males	
	ASR	SRR^a^	ASR	SRR^a^	ASR	SRR^a^
2008	0.09	0.00 (0.00–0.00)	0.19	0.01 (0.00–0.01) ^b^	0.00	0.00 (0.00–0.00)
2009	2.61	0.10 (0.09–0.10) ^b^	5.26	0.18 (0.17–0.18) ^b^	0.07	0.00 (0.00–0.00)
2010	5.31	0.20 (0.19–0.20) ^b^	10.12	0.35 (0.34–0.35) ^b^	0.79	0.03 (0.03–0.03) ^b^
2011	9.24	0.34 (0.34–0.35) ^b^	14.24	0.49 (0.48–0.49) ^b^	4.61	0.18 (0.18–0.19) ^b^
2012	14.66	0.54 (0.53–0.55) ^b^	19.28	0.66 (0.65–0.67) ^b^	10.40	0.42 (0.41–0.42) ^b^
2013	18.24	0.67 (0.67–0.68) ^b^	22.53	0.77 (0.76–0.78) ^b^	14.27	0.57 (0.56–0.58) ^b^
2014	21.99	0.81 (0.80–0.82) ^b^	26.20	0.90 (0.88–0.91) ^b^	17.97	0.72 (0.71–0.73) ^b^
2015	25.09	0.93 (0.92–0.94) ^b^	28.39	0.97 (0.96 (0.98) ^b^	21.94	0.88 (0.86–0.89) ^b^
2016	27.71	1.02 (1.01–1.03) ^b^	30.27	1.03 (1.02–1.05) ^b^	25.26	1.01 (1.00–1.03) ^b^
2017	27.06	1.00 (Reference)	29.26	1.00 (Reference)	24.96	1.00 (Reference)
2018	27.80	1.03 (1.02–1.04) ^b^	29.73	1.02 (1.00–1.03) ^b^	25.95	1.04 (1.02–1.06) ^b^
2019	29.07	1.07 (1.06–1.09) ^b^	30.71	1.05 (1.03–1.07) ^b^	27.49	1.10 (1.08–1.12) ^b^

^a^ The ratio of each year ASR to 2017 ASR as reference, with 95% confidence interval in parentheses.

^b^ P-value < 0.05

## Discussion

Our study found significant evidence of improvement in vaccination rates associated with the HPV mandatory school-entry vaccination policy. One year after implementation, adolescents from 11 to 12 years old, whom the mandate applied at implementation, began to lead initiation rates (89.8%) compared to adolescents 13 to 17 years (82.6%). Therefore, our findings in HPV vaccine initiation coverage rates adds evidence that the way the school-entry policy is designed and implemented impact HPV vaccination uptake. PR immunization law (Law 25 of 1983) only allows opt-out for religious and medical reasons, reinforcing the idea that limited opt-out policies may be more effective in promoting vaccine uptake [14, 27]. In addition, at the time of this study, only one dose of the HPV vaccine series was required. For the 2021–2022 school year in PR, the new HPV vaccination requirement includes completing recommended dose series; this could impact UTD vaccine coverage rates. Improvements in HPV vaccine initiation coverage associated with an HPV policy implementation were also evident after one year of enactment in Rhode Island [14].

An associated increase in HPV UTD vaccine coverage was observed. We found a raw UTD vaccine coverage rate of 21.5% among adolescents 11 to 12 years in 2019 and a significant increase of 7% in the same year, compared to the reference year of 2017. However, even when significant improvement is evidenced, an overall low HPV UTD vaccine coverage rate can be observed. When we compared our findings with NIS Teen UTD coverage estimates reported for 2016 and 2019 (data are not presented for PR in 2017 and 2018), these were relative higher than the results reported in our analysis, showing a difference of 19.1% for 2016 (NIS 2016: 52.8% vs. PRIR: 33.7%), and a difference of 26.3% for 2019 (NIS 2019: 58.4% vs. PRIR 2019: 32.1%) [43]. Observed differences may be due to various reasons regarding the way data is collected. NIS Teen coverage estimates are from teens with adequate provider data; data collected from the teen vaccine provider(s) [44, 45]. A first household interview to parents is done to screen teen vaccination status. Parents then consent to interviewers to verify the immunization status of the teens with their vaccine provider (s). In 2016, teens with adequate provider data percentage (%) was 34.3%, while in 2019, it was 25.9% [44, 45]. Therefore, low response rates observed in NIS-Teen data could potentially limit the generalizability of these findings. Besides, there is also the possibility that the parents who gave consent could also be more engaged with the vaccines topic and more willing to endorse the completion of recommended doses than those who did not. On the other hand, suboptimal UTD vaccine coverage rates found in our study could be explained by the design and the way the requirement was implemented; at the moment this study was conducted, only one dose of HPV was required for school-entry [46]. However, all HPV vaccine coverage rates showed an improvement after the implementation of the policy, which provides additional evidence that school-entry policies can be an effective tool for increasing childhood vaccination rates [14, 25, 26].

When evaluating the entire cohort, the gap in rates between sexes closes throughout the observed period. Thus, we can suggest that implemented policy could have led to a minimal difference in HPV rates at the end of the period, further reducing sex disparities in HPV vaccine initiation coverage [14]. When observing the entire period, trends in UTD vaccine coverage between adolescents 11 to 12 years also shows how the gap in rates between sexes dissipates at the end of the period.

Previous studies have documented that parents, primarily *Latino* or Spanish-speaking parents, perceive that the age of 11 is too early for HPV vaccination and also express concern that this could promote sexual activity [47–49]. Hence, this explains that for most of the period before implementation, most of those who initiated were between 13 to 17 years old. Although we observe that adolescents aged 13 to 17 years also lead HPV UTD vaccine coverage rates, it is important to highlight that the UTD vaccine coverage rates of adolescents between 11 and 12 years began to improve after policy implementation.

The period from 2008 to 2010 (or 2011) shows the lowest raw and adjusted rates. The recent authorizations and recommendations for HPV vaccination, for that moment, promoted these early improvements. The FDA authorized the quadrivalent HPV vaccine in 2006 for women 9 to 26 years of age [5]. In the same year, ACIP recommended the same vaccine for female adolescents 11 to 12 years for routine vaccination and females 13 to 26 years for catch-up vaccination [5]. FDA authorized the quadrivalent vaccine for males in 2009 [6]. In 2011, ACIP expanded the recommendations to male adolescents from 11 to 12 years for routine vaccination and catch-up vaccination for males 13 to 21 years old [6]. These catch-up recommendations for males have been extended to 26 years old [50]. In addition to the ACIP recommendation, this increase might be attributed to assertive efforts from local coalitions [51] and community-based organizations during these early days [17].

An important consideration for this analysis was the high migratory observed in the last years in PR reason why this study performed standardized rates to control population changes. Overall, and stratified by sex, SRRs showed significant excesses in initiation rates and UTD vaccine coverage after policy implementation. These findings confirmed that the mandate affected both the initiation rates and UTD vaccine coverage, even with the high migration in PR.

Results in this study should consider possible limitations. The obtained data for this study includes adolescents immunized with at least one of the following vaccines: against HPV, influenza, influenza A, tetanus, Td, Tdap, meningococcal conjugate, and meningococcal serogroup B. This limitation could induce an estimation bias in the analysis; hence, a greater number of adolescents could also have been included in the denominator, affecting the specific value of these rates. However, this possibility would not affect the observed rate per year trend found in this study. Despite possible limitations, this study used data from PRIR IIS, thus reducing the possibility of information bias. We also performed standardized rates by age, which also provided us with information on the impact of the policy, controlling for the effect of age for possible changes in the population structure.

Our study evaluated the impact of the HPV school entry policy after one year of enactment; subsequent analyses must assess how the policy continues affecting vaccine coverage afterward and compares with the coverage of other vaccines already part of the curriculum for school entry. Consideration should be given to assess how HPV coverage may have been affected by the recent COVID-19 pandemic.

## Supporting information

S1 File Tables A-C. (DOCX)
