# Supplementary material for: Human papillomavirus vaccine initiation and up-to-date vaccine coverage for adolescents after the implementation of school-entry policy in Puerto Rico
Source: PLOS Glob Public Health. 2022 Nov 8;2(11):e0000782. doi: 10.1371/journal.pgph.0000782 (PMC10021633; doi:10.1371/journal.pgph.0000782)
Supplement: S1 File — (DOCX) [file pgph.0000782.s001.docx]

Table A. Distribution of population 11 to 17 years by sex from 2008 to 2019

| **Year** | **Females** | | | **Males** | | |
| --- | --- | --- | --- | --- | --- | --- |
|  | **X** | **Y** | **N** | **X** | **Y** | **N** |
| 2008 | 7,101 | 197 | 78,155 | 139 | 1 | 81,153 |
| 2009 | 24,814 | 5,314 | 93,496 | 550 | 80 | 97,773 |
| 2010 | 37,383 | 10,443 | 105,564 | 13,019 | 900 | 111,805 |
| 2011 | 57,709 | 15,833 | 114,576 | 38,862 | 5,684 | 122,954 |
| 2012 | 68,677 | 22,658 | 119,324 | 54,760 | 13,278 | 128,482 |
| 2013 | 76,865 | 27,913 | 123,111 | 65,171 | 19,053 | 132,135 |
| 2014 | 83,692 | 33,504 | 125,440 | 73,269 | 24,074 | 131,492 |
| 2015 | 86,528 | 35,840 | 123,307 | 80,524 | 28,904 | 128,821 |
| 2016 | 86,345 | 37,025 | 119,037 | 83,954 | 32,390 | 124,589 |
| 2017 | 83,956 | 34,556 | 113,328 | 83,254 | 30,851 | 118,305 |
| 2018 | 82,685 | 32,103 | 102,936 | 83,470 | 29,087 | 106,857 |
| 2019 | 75,349 | 28,953 | 89,302 | 76,654 | 26,668 | 92,467 |

Table B. Distribution of population 11 to 12 years old overall and by sex from 2008 to 2019

|  | **Overall** | | | | **Females** | | | **Males** | | |
| --- | --- | --- | --- | --- | --- | --- | --- | --- | --- | --- |
| **Year** | **X** | **Y** | **N** | **X** | | **Y** | **N** | **X** | **Y** | **N** |
| 2008 | 5,456 | 184 | 68,121 | 5,345 | | 183 | 31,590 | 111 | 1 | 36,531 |
| 2009 | 12,833 | 3,052 | 74,357 | 12,529 | | 3,000 | 36,377 | 304 | 50 | 37,980 |
| 2010 | 18,783 | 3,259 | 76,342 | 12,761 | | 2,867 | 37,485 | 6,022 | 392 | 38,857 |
| 2011 | 32,436 | 4,960 | 78,973 | 17,583 | | 3,018 | 38,390 | 14,853 | 1942 | 40,583 |
| 2012 | 33,079 | 6,499 | 76,116 | 17,443 | | 3,670 | 37,054 | 15,636 | 2829 | 39,062 |
| 2013 | 31,169 | 6,020 | 70,543 | 16,451 | | 3,385 | 34,530 | 14,718 | 2635 | 36,013 |
| 2014 | 32,573 | 6,385 | 67,967 | 16,881 | | 3,556 | 33,258 | 15,692 | 2829 | 34,709 |
| 2015 | 35,279 | 7,441 | 64,979 | 17,938 | | 4,023 | 31,770 | 17,341 | 3418 | 33,209 |
| 2016 | 35,138 | 7,861 | 61,051 | 17,721 | | 4,185 | 29,974 | 17,417 | 3676 | 31,077 |
| 2017 | 32,583 | 5,184 | 55,847 | 16,313 | | 2,748 | 27,506 | 16,270 | 2436 | 28,341 |
| 2018 | 34,407 | 6,574 | 45,087 | 16,957 | | 3,418 | 22,252 | 17,450 | 3156 | 22,835 |
| 2019 | 23,541 | 5,636 | 26,215 | 11,645 | | 2,880 | 13,003 | 11,896 | 2756 | 13,212 |

Table C. Distribution of population 13 to 17 years old overall and by sex from 2008 to 2019

|  | **Overall** | | | **Females** | | | **Males** | | | |
| --- | --- | --- | --- | --- | --- | --- | --- | --- | --- | --- |
| **Year** | **X** | **Y** | **N** | **X** | **Y** | **N** | **X** | **Y** | **N** |  |
| 2008 | 1,784 | 14 | 91,187 | 1,756 | 14 | 46,565 | 28 | 0 | 44,622 |  |
| 2009 | 12,531 | 2,342 | 116,912 | 12,285 | 2,314 | 57,119 | 246 | 28 | 59,793 |  |
| 2010 | 31,619 | 8,084 | 141,027 | 24,622 | 7,576 | 68,079 | 6,997 | 508 | 72,948 |  |
| 2011 | 64,135 | 16,557 | 158,557 | 40,126 | 12,815 | 76,186 | 24,009 | 3742 | 82,371 |  |
| 2012 | 90,358 | 29,437 | 171,690 | 51,234 | 18,988 | 82,270 | 39,124 | 10449 | 89,420 |  |
| 2013 | 110,867 | 40,946 | 184,703 | 60,414 | 24,528 | 88,581 | 50,453 | 16418 | 96,122 |  |
| 2014 | 124,388 | 51,193 | 188,965 | 66,811 | 29,948 | 92,182 | 57,577 | 21245 | 96,783 |  |
| 2015 | 131,773 | 57,303 | 187,149 | 68,590 | 31,817 | 91,537 | 63,183 | 25486 | 95,612 |  |
| 2016 | 135,161 | 61,554 | 182,575 | 68,624 | 32,840 | 89,063 | 66,537 | 28714 | 93,512 |  |
| 2017 | 134,627 | 60,223 | 175,786 | 67,643 | 31,808 | 85,822 | 66,984 | 28415 | 89,964 |  |
| 2018 | 131,748 | 54,616 | 164,706 | 65,728 | 28,685 | 80,684 | 66,020 | 25931 | 84,022 |  |
| 2019 | 128,462 | 49,985 | 155,554 | 63,704 | 26,073 | 76,299 | 64,758 | 23912 | 79,255 |  |

*X= Persons in certain age range with at least 1 dose as of Dec 31 per year*

*Y= Persons in certain age with recommended doses as of Dec 31 per year*

*N= Population in certain age range as of Dec 31 per year*

The vaccine coverage calculation can be made with the formulas previously presented:

$HPV Vaccine Initiation Coverage= \frac{X}{N}$ ** 100*

$HPV UTD Vaccine Coverage= \frac{Y}{N}$ ** 100*
